# Immobilization of Pseudorabies Virus in Porcine Tracheal Respiratory Mucus Revealed by Single Particle Tracking

**DOI:** 10.1371/journal.pone.0051054

**Published:** 2012-12-07

**Authors:** Xiaoyun Yang, Katrien Forier, Lennert Steukers, Sandra Van Vlierberghe, Peter Dubruel, Kevin Braeckmans, Sarah Glorieux, Hans J. Nauwynck

**Affiliations:** 1 Laboratory of Virology, Department of Virology, Parasitology and Immunology, Faculty of Veterinary Medicine, Ghent University, Salisburylaan, Merelbeke, Belgium; 2 Laboratory of General Biochemistry and Physical Pharmacy, Ghent University, Harelbekestraat, Ghent, Belgium; 3 Center for Nano- and Biophotonics, Ghent University, Harelbekestraat, Ghent, Belgium; 4 Polymer Chemistry & Biomaterials Group, Ghent, Belgium; Queen’s University, Canada

## Abstract

Pseudorabies virus (PRV) initially replicates in the porcine upper respiratory tract. It easily invades the mucosae and submucosae for subsequent spread throughout the body via blood vessels and nervous system. In this context, PRV developed ingenious processes to overcome different barriers such as epithelial cells and the basement membrane. Another important but often overlooked barrier is the substantial mucus layer which coats the mucosae. However, little is known about how PRV particles interact with porcine respiratory mucus. We therefore measured the barrier properties of porcine tracheal respiratory mucus, and investigated the mobility of nanoparticles including PRV in this mucus. We developed an *in vitro* model utilizing single particle tracking microscopy. Firstly, the mucus pore size was evaluated with polyethylene glycol coupled (PEGylated) nanoparticles and atomic force microscope. Secondly, the mobility of PRV in porcine tracheal respiratory mucus was examined and compared with that of negative, positive and PEGylated nanoparticles. The pore size of porcine tracheal respiratory mucus ranged from 80 to 1500 nm, with an average diameter of 455±240 nm. PRV (zeta potential: −31.8±1.5 mV) experienced a severe obstruction in porcine tracheal respiratory mucus, diffusing 59-fold more slowly than in water. Similarly, the highly negatively (−49.8±0.6 mV) and positively (36.7±1.1 mV) charged nanoparticles were significantly trapped. In contrast, the nearly neutral, hydrophilic PEGylated nanoparticles (−9.6±0.8 mV) diffused rapidly, with the majority of particles moving 50-fold faster than PRV. The mobility of the particles measured was found to be related but not correlated to their surface charge. Furthermore, PEGylated PRV (-13.8±0.9 mV) was observed to diffuse 13-fold faster than native PRV. These findings clearly show that the mobility of PRV was significantly hindered in porcine tracheal respiratory mucus, and that the obstruction of PRV was due to complex mucoadhesive interactions including charge interactions rather than size exclusion.

## Introduction

Herpesviruses are double-stranded DNA viruses with the potential to cause severe diseases in different species. Many members of the subfamily *Alphaherpesvirinae* initially replicate in the epithelial cells of the respiratory and/or genital mucosae [1]–[3]. An important member is Pseudorabies Virus (PRV), the prototype veterinary alphaherpesvirus. PRV causes respiratory tract problems, nervous system disorders and abortions in pigs. After local replication in the epithelial cells, these viruses gain access to the connective tissue and find their way to blood vessels and nerve endings for spread throughout the host [4]–[7]. Different important barriers of the host try to hamper PRV invasion. Recently, it has been shown that in order to invade, different alphaherpesviruses including PRV use proper processes to pass an important barrier underneath the epithelium, the basement membrane [2], [8], [9]. Importantly, prior to epithelial cell entry, a mucus layer that coats the epithelium and serves as the first line of defense, has to be overcome by the virus [10].

Mucus is a viscoelastic and adhesive gel that coats the non-keratinized epithelial surface of different mucosae. The thickness of the mucus layer varies among different types and species, ranging from several to more than 100 microns for the trachea of different animals [11]–[13]. Next to serving as a lubricant and assisting the adsorption of nutrients, the mucus layer acts as the body’s first barrier against microbial infections. The major component and property determinant are heavily glycosylated glycoproteins, known as mucins. The assembly of these mucins in fiber structures creates an entangled mucus meshwork [14], [15]. The mesh (pore) size sets a threshold beyond which particle diffusion is hindered: particles with diameters larger than this mesh size are rejected while smaller, minimally interactive particles should pass through the mucus. However, in addition to this size filtering mechanism, mucus seems to use more sophisticated strategies to interact with the traversing particles, especially diverse viruses that attempt to invade the body through the mucosae. Olmsted et al. [16] reported that two capsid virus-like particles, human papillomavirus (diameter 55 nm) and Norwalk virus (38 nm), diffused rapidly in human cervicovaginal mucus, because of the neutral charge of viral capsids and probably also due to the size. It is hypothesized that small particles expose less hydrophobic patches, and are therefore limited in making polyvalent bonds with the hydrophobic domains distributed along the mucin fibers. However, Hida and colleagues described an even smaller capsid virus, adeno-associated virus serotype 5 (20 nm), to be highly trapped in human cystic fibrosis sputum of which the pore size ranged from 60 to 300 nm [17]. To date, the motion of different viruses ranging from large enveloped viruses, such as herpes simplex virus [18] and human immunodeficiency virus (HIV) [19], to small non-enveloped viruses, such as adenovirus [17], human papillomavirus and adeno-associated virus, have been investigated in mucus. However, these studies were limited to human cervicovaginal mucus and human cystic fibrosis sputum because none of the other types of mucus were readily available. Particularly, the interaction of aerosol respiratory viruses with healthy respiratory mucus has never been studied. Unraveling the mechanism behind the penetration of viruses across the mucosal barriers has potentially significant implications for the development of novel antiviral strategies. To achieve a better understanding in this matter, an *in vitro* model resembling the *in vivo* situation is needed. As PRV is commonly used as a good model for human alphaherpesviruses, it is highly interesting to investigate how PRV interacts with porcine respiratory mucus. Therefore, the main objectives of this study were to fully characterize the filtering properties of porcine respiratory mucus to nanoparticulate systems, and to better understand the interactions between PRV and the mucus through which it invades in an attempt to unravel the mechanism of how PRV penetrates through porcine respiratory mucus.

In order to measure the microstructure of porcine respiratory mucus, atomic force microscopy (AFM), a high-resolution imaging technique, was used to visualize the surface of the mucus samples and to measure the pore size formed by the mucus elements. It enables to measure the forces acting between a sample and a fine tip, which is attached to the free end of a cantilever and brought very close to the surface. Attractive or repulsive forces resulting from interactions between the tip and the surface can lead to a deflection of the cantilever according to Hooke’s law [20], [21]. As such, a three-dimensional image of the sample surface can be obtained and the pore size can be measured with the line profiling feature of the AFM software. Additionally, the effective pore size of porcine tracheal respiratory mucus was further evaluated by analyzing the motion of polyethylene glycol (PEG) coupled, non-adhesive nanoparticles in function of particle size. Based on the theory that particle transport in heterogeneous environments is regulated by the local properties of the material, non-adhesive particles smaller than the effective mesh spacing (pore size) of the network are capable of diffusion because they tend to experience the lower viscosity of the interstitial space [22], [23].

In this study, we have developed a model to study the virus-mucus interactions and demonstrated the mucoadhesive influence of porcine respiratory mucus on the mobility of PRV. We have implied single particle tracking (SPT) microscopy, which uses high speed video microscopy to track the diffusion of hundreds of particles simultaneously and analyzes the motion of particles. Particle trajectories are used to calculate time-averaged mean squared displacements (*MSD*) and to determine time-dependent diffusion coefficients, which are subsequently used to characterize individual particle transport modes [24]. Utilizing the technique, we analyzed the microstructure of the porcine tracheal respiratory mucus, and detected the mobility of PRV and different charge modified nanoparticles in the mucus.

## Materials and Methods

### Mucus Sampling and Preparation

Tracheal respiratory mucus was collected from porcine tracheas within 3 h after slaughter. Porcine tracheas were obtained with the permission of and collected from G. Van Landschoot & Zonen N.V., Adegem, Belgium (a local slaughterhouse) and stored on ice prior to mucus collection. The trachea was isolated and cut open longitudinally. Mucus was gently scraped with a spoon, collected with a syringe, and stored at –70°C until use.

### Atomic Force Microscopy

Porcine tracheal respiratory mucus (100 µl) was evenly coated on glass (1 cm^2^) and was air-dried at room temperature, according to the protocol adapted from Broughton-Head et al. [25]. Atomic force microscopy (AFM) images were obtained in ambient conditions in air using a multimode scanning probe microscope (Digital Instruments, Santa Barbara, CA, USA) equipped with a Nanoscope IIIa controller. 5 µm×5 µm scans were recorded in tapping mode using a silicon cantilever (OTESPA, Veeco, CA, USA). Pore diameters were obtained using the line profiling feature of the AFM software (NanoScope software version 4.43r8, Veeco Instruments, USA).

### Preparation and Characterization of Nanoparticles

Negatively charged, yellow-green fluorescent carboxylate-modified FluoSpheres® of different diameters, 100 nm, 200 nm and 500 nm, were purchased from Invitrogen (Merelbeke, Belgium). According to the protocol of Symens et al. [26], positively charged nanoparticles were obtained as a result of amide formation between the carboxylate groups of the carboxylate modified FluoSpheres® and the primary amine group of N, N-dimethylethylenediamine. PEGylated nanoparticles were prepared by covalent modification of the surface carboxylate groups of carboxylate modified FluoSpheres® with methoxy-polyethylene glycol-amine (mPEGa, 2 kD, Creative PEGWorks, Winston Salem, USA.) as previously described [27]. Particle size (diameter) and surface charge (zeta potential) were determined by dynamic light scattering and laser Doppler anemometry, respectively, using the Zetaziser Nano-ZS (Malvern, Worcestershire, UK).

### PEGylation of PRV

The recombinant PRV Becker strain expressing green fluorescent protein fused to the VP26 capsid protein was a kind gift of Dr. Greg Smith (Northwestern University, Chicago, IL, USA). The PRV stock (10^8.0^ TCID_50_/ml) was concentrated by ultracentrifugation at 80 000 g for 90 min at 4°C in a Type 35 rotor (Beckman, Fullerton, CA, USA). The pellet was resuspended in phosphate buffered saline (PBS: 137 mM NaCl, 10 mM Na_2_HPO_4_, 2.7 mM KCl, 2 mM KH_2_PO_4,_ pH 7.4), loaded upon a 30% sucrose cushion and followed by ultracentrifugation at 100 000 g for 3 h at 4°C in an SW41Ti rotor (Beckman, Fullerton, CA, USA). The virus pellet was resuspended in PBS to give an approximate concentration of 10^10^ TCID_50_/ml. Methoxy-polyethylene glycol activated by succinimidyl succinate (mPEG-NHS, 2 kD) (NANCOS, Huissen, Netherlands) was added to the virus suspension, giving a final concentration of 20 mg/ml. The coupling reaction was performed for 2 h at 25°C. Afterwards, unreacted mPEG-NHS and the byproducts were removed by buffer exchange over a centrifugal filter (50 K membrane, Millipore). The size and zeta (ζ) potential of the PEGylated PRV were measured with the Zetaziser Nano-ZS (Malvern) and the PEGylated PRV was preserved at 4°C until use.

### Real-time Single Particle Tracking

The trajectories of fluorescent particles in porcine tracheal respiratory mucus were recorded by a fast and sensitive electron-multiplying charge-coupled device (EMCCD) camera (Cascade II: 512; Roper Scientific, Tucson, AZ, USA) mounted on an inverted epifluorescence microscope (Nikon TE2000E, Nikon Belux, Brussels, Belgium) equipped with a 100× oil-immersion objective (Plan Apochromat, Nikon). Tracking experiments were performed in press-to-seal™ silicone isolators (20 mm diameter, 0.5 mm deep, Invitrogen, Merelbeke, Belgium). Three microliters of virus suspension (10^10^ TCID_50_/ml) or nanoparticles (0.004 wt.-%) were mixed with 100 µl porcine tracheal respiratory mucus by gentle stirring to avoid perturbation. The movies were captured with the NIS Elements AR software (Nikon) at a temporal resolution of 46.2 ms for 5 s. The illumination time was 10 ms per frame. Trajectories of n ≥500 particles were analyzed for each experiment and at least three independent experiments were performed for each condition. Movies were analyzed with the Image Processing Software (IPS, in-house developed software) to extract x, y positional data over time. The time-averaged mean squared displacements (*MSD*) and apparent diffusion coefficient (*D_a_*) were calculated as a function of the time scale (*t*) for each particle.

### Data Analysis

Analysis of the movies was performed with custom made image processing software [28]. Individual particles were identified in each frame of a movie and their centroids were calculated. The trajectories of the particles were determined by a nearest neighbor algorithm. For each trajectory, the mean squared displacement (*MSD*) was calculated for the available time lags *t* (i.e. multiples of the time Δ*t* between the images). The *MSD* versus *t* plots were analyzed by a weighted fit of the anomalous diffusion model [29], [30]: *MSD* = *Γt^α^*, with the transport coefficient *Γ* and the anomalous exponent *α* as free fitting parameters. The value of *α* is a measure for the mode of diffusion: for free diffusion *α* = 1 (*Γ* becomes equal to 4 times the diffusion coefficient), while *α* <1 indicates hindered diffusion. The deviation of *α* from 1 is thus a measure for the anomaly of the diffusion. By analyzing the trajectories according to this anomalous diffusion model, distributions of the corresponding *α*-values can be obtained. Besides this, the apparent diffusion coefficient *D_a_* corresponding to the first time lag Δ*t* was calculated according to the classical expression: *D_a_* = *MSD/*4Δ*t* [30].

Note that in the case of free diffusion, *D_a_* reduces to the diffusion coefficient. Distributions of the apparent diffusion coefficient can be obtained by analyzing the trajectories of the particles. These distributions are further refined with a maximum entropy method (MEM) [28]. The MEM analysis improves the precision of the distributions, and removes features that are not statistically warranted by the data.

## Results

### Microstructure of Porcine Tracheal Respiratory Mucus

**Figure 1:**
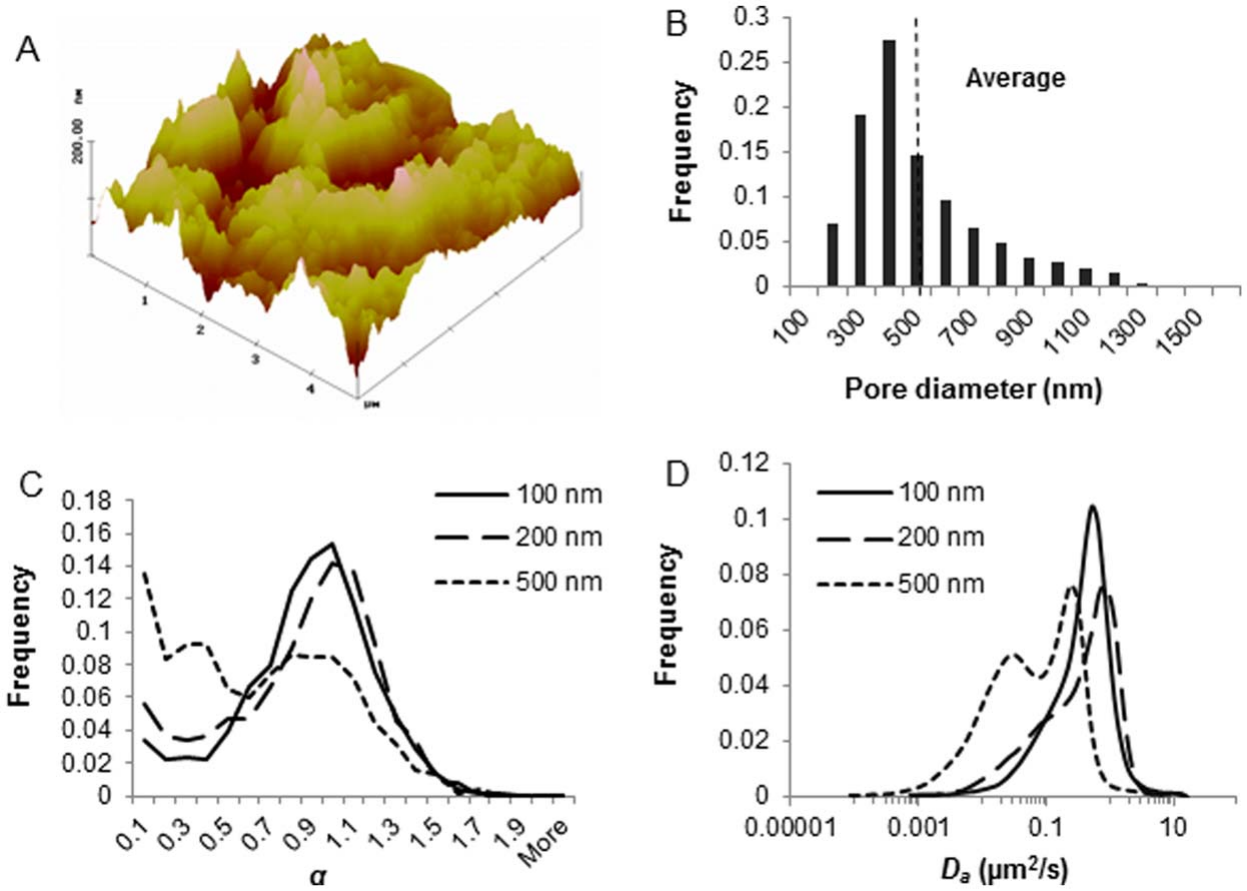
Evaluation of the microstructure of porcine tracheal respiratory mucus. (A) AFM visualization (5 µm×5 µm) of porcine tracheal respiratory mucus. (B) Pore size distribution of the porcine tracheal respiratory mucus as measured by AFM. Pore diameters were analyzed by the line profiling of the 3-D images. Three scans were performed for each sample, and three independent samples were measured. The dashed line indicates the average of pore diameters (n = 500). (C) Transport modes of 100, 200 and 500 nm PEGylated nanoparticles indicated by *α* value. More than 2000 trajectories from 3 independent experiments were tested to obtain *α*. (D) Distributions of the apparent diffusion coefficient of PEGylated nanoparticles. Trajectories of 10 steps were analyzed for each of the 2000 diffusion coefficients. Distributions were refined with MEM.

The porous nature of porcine tracheal respiratory mucus was visualized using TappingMode AFM. The color intensity shows the vertical profile of the sample surface, with light regions representing the highest points and the dark points representing the depressions and pores (Figure 1A). Pore diameters were measured with the line profiling feature of the AFM software. Distribution of the pore diameters (n = 500) obtained from three independent experiments illustrated that the pore size of the porcine tracheal respiratory mucus was highly various, ranging between 80 and 1500 nm. The average pore size was 455±240 nm in diameter, with 85% of the pores being larger than the diameter of PRV particles (Figure 1B). To further evaluate the effective mesh spacing of porcine tracheal respiratory mucus, translocations of 100, 200, and 500 nm PEGylated muco-innert nanoparticles, were tracked and transport rates were analyzed. The *MSD* of the nanoparticles was fitted to the equation *MSD = Γ*Δ*t^α^* to obtain *α*, an indicator of the extent of particle impediment. After PEGylation, the nanoparticles were slightly enlarged in diameter due to the coverage with PEG. The nearly neutral surface charge suggested that the PEGylation of the particles was complete (Table 1). As demonstrated in Figure 1C, most of 100 nm and 200 nm PEGylated nanoparticles diffused freely through mucus, whereas, part of the 500 nm PEGylated nanoparticles were subdiffusive. This partially hindered motion is also apparent from the bimodality of the distribution of diffusion coefficient for the 500 nm nanoparticles (Figure 1D).

**Table 1:** Surface charge and size of the PEGylated nanoparticles.

Size	ζ potential (mV)	Diameter (nm)
100 nm*	−8.7±0.5	124.6±0.5
200 nm*	−9.6±0.8	232.6±2.4
500 nm*	−9.3±0.9	538.0±7.5

* Provided by the manufacture.

### PRV was Highly Obstructed in Porcine Tracheal Respiratory Mucus

**Figure 2:**
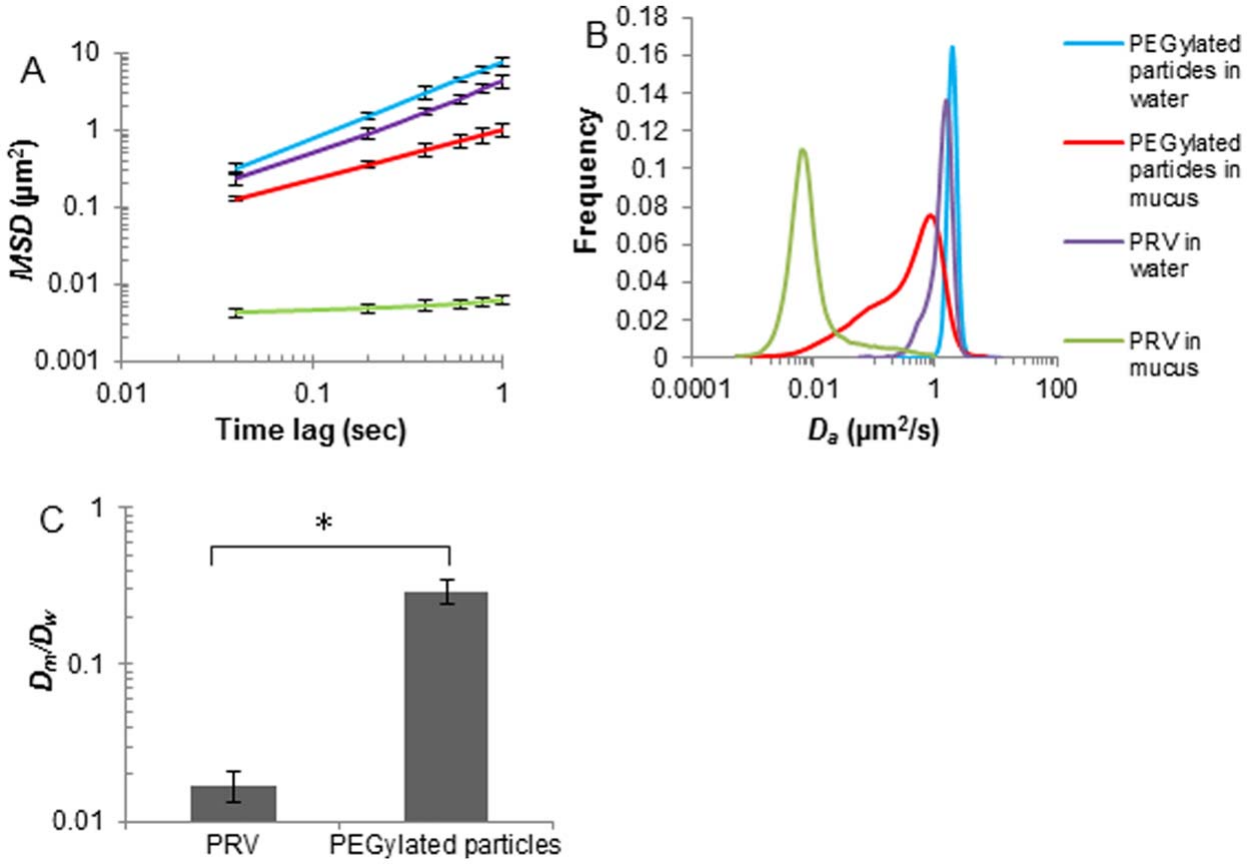
Transport rates of PRV and 200 nm PEGylated nanoparticles in porcine tracheal respiratory mucus or in water. (A) Averaged ensemble mean squared displacements <*MSD*> of PRV and 200 nm PEGylated nanoparticles with respect to time scale. Error bars indicate standard errors of the mean. (B) Distributions of the apparent diffusion coefficient of PRV and 200 nm PEGylated nanoparticles. Trajectories of 10 steps were analyzed for each of the 2000 diffusion coefficients. Distributions were refined with MEM. (C) Ratios of the average diffusion coefficients in mucus (*D_m_*) to those in water (*D_w_*). Three independent experiments were performed for each condition. Error bars indicate the standard deviation. The asterisk (*) indicates statistical significance (P<0.005).

The motion of PRV (245.7±11.5 nm) in porcine tracheal respiratory mucus or in water was investigated and compared with those of 200 nm PEGylated nanoparticles. PRV was greatly slowed down in mucus with respect to the PEGylated particles. At the time scale of 1 s, the ensemble mean squared displacement <*MSD*> of the PEGylated nanoparticles was 160-fold higher than that of PRV (Figure 2A). Trajectories of 10 steps have been analyzed, from which a distribution of the apparent diffusion coefficients was obtained (Figure 2B). This clearly demonstrates that PRV experienced a severe obstruction in porcine tracheal respiratory mucus, with the majority of virus particles diffusing 50-fold slower than PEGylated nanoparticles. Similarly, an average *α* value of 0.17 was observed for PRV compared to 0.86 for the 200 nm PEGylated nanoparticles. PEGylated nanoparticles thus freely diffused through the porcine tracheal respiratory mucus whereas PRV was significantly immobilized.

### Particle Surface Charge and Particle Mobility

**Figure 3:**
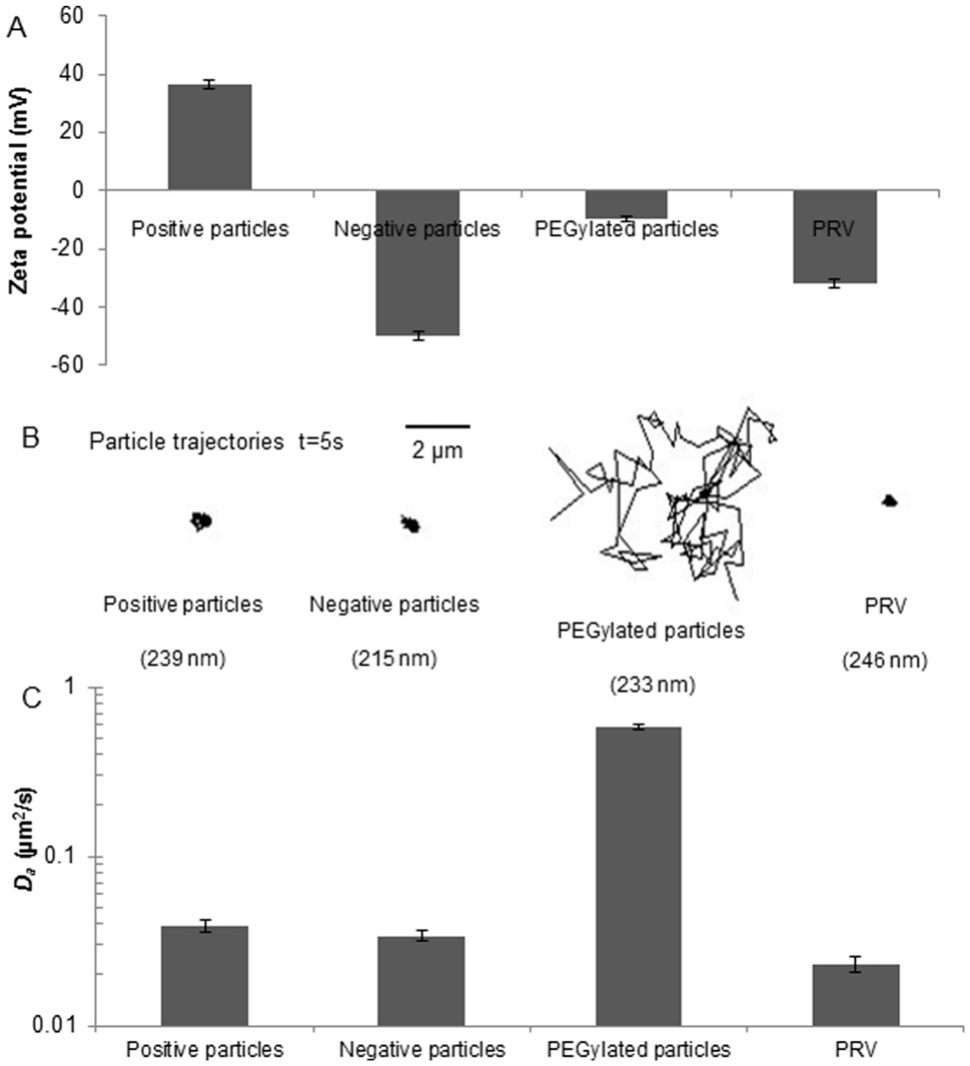
Particle surface charge and particle mobility. (A) ζ potentials for the particles measured with laser Doppler anemometry. (B) Representative trajectories of PRV and 200 nm nanoparticles with different surface charge modifications. The diffusions of negatively and positively charged nanoparticles and PRV were significantly suppressed; the PEGylated nanoparticles diffused rapidly. (C) Diffusion coefficient of the PRV and modified nanoparticles. Apparent diffusion coefficients *D_a_* were determined by particle tracking for ensembles of 2000 particles for each condition. Error bars indicate the standard error of the mean.

To determine the relationship between surface charge and particle mobility, the diffusion of strongly negatively and positively charged nanoparticles, the nearly neutral, hydrophilic PEGylated nanoparticles (200 nm) and PRV were measured in mucus. Indicated by the representative trajectories (Figure 3B), the diffusive motion of the strongly charged nanoparticles and PRV was highly obstructed. In contrast, PEGylated nanoparticles were allowed to diffuse rapidly. PEGylated nanoparticles diffused 30-fold faster than PRV in mucus and more than 15 and 17-fold faster than the positive and negative nanoparticles, respectively (Figure 3C). These data suggest that the surface charge was involved in the interactions between particles and the mucus environments, which further affected the particle mobility. However, PRV clearly diffused more slowly than the higher negatively charged nanoparticles implying that other types of interactions may also play a role in this case.

### PRV Mobility was Increased by PEGylation

The PEGylated PRV was less adhesive to porcine tracheal respiratory mucus than the native PRV. After PEGylation, the diameter of PEGylated PRV was slightly greater than the uncoated virus. ζ potential analysis revealed that the surface charge was altered from −31.8±1.5 mV to −13.8±0.9 mV after PEGylation. The transport rate of PEGylated PRV was 11-fold higher than PRV (Table 2). Taken together, the mobility of PRV in porcine tracheal respiratory mucus was increased by coating uncharged, hydrophilic PEG onto its surface.

**Table 2:** Diffusion comparison of PRV and PEGylated PRV.

Particles	*α*	Diameter (nm)	ζ potential (mV)	*D_a_* (µm^2^/s)	*D_m_*/*D_w_*
PRV	0.167	245.7±11.5	−31.8±1.5	0.023±0.061	0.017±0.004
PEGylated PRV	0.591	269.4±16.3	−13.8±0.9	0.289±0.779	0.212±0.182

## Discussion

PRV, often used as a representative for alphaherpesviruses, initially infects the porcine upper respiratory tract. In order to unravel the invasion of PRV through the mucus layer, we have set up an *in vitro* model of porcine tracheal respiratory mucus and analyzed the mobility of PRV through it.

In the first part, AFM was used to visualize and measure the pore diameter in porcine tracheal respiratory mucus. AFM has several advantages over electron microscopy. Firstly, analysis of the cantilever deflections enables the calculation of all three dimensions of the currently analyzed feature of the specimen, e.g. width, length and height. As a result, three-dimensional images are generated [31]. Secondly, samples viewed by AFM do not require special treatments such as fixation and metal coating that would irreversibly change or damage the samples. Thirdly, when imaging a dried sample deposited on a glass surface instead of a hydrated sample, the mucus polymers are immobilized, and the resolution is improved [32], [33]. Using this technique, we demonstrated that the mesh size of porcine tracheal respiratory mucus was highly heterogeneous, ranging from 80 nm to 1500 nm. Furthermore, PEGylated nanoparticles are minimally interactive with mucus and therefore are an ideal approach to probe the mucus microscopic structure. The dense PEG shielding is able to provide nanoparticles with highly hydrophilic surface properties, resulting in less hydrophobic patches subjected to mucoadhesion as well as less capability of ionic bonding due to the nearly uncharged surface (PEG is nonionic at physiological pH values). The hindered motion of the 500 nm PEGylated nanoparticles suggested that these particles were probably obstructed by the mesh. This also fits the pore size estimation by AFM. Unlike mucus, the range of the pore size of the collagen-laminin network in the basement membrane is much smaller, approximately 50–100 nm [34]–[36]. Only small particles are able to diffuse across such pores. Passage of microorganisms through this barrier often involves regulatory protease breakdown of the collagen-laminin network. Thus the basement membrane clearly warrants a potent physical barrier against pathogenic invasion and passage of microorganisms. As the mucus pore size is tremendously heterogeneous, and most of the pores are larger than the diameter of viruses, it would be risky if the mucus exclusively relies on a size exclusion mechanism to repel the potentially invading viruses. Instead, alternative filtering mechanisms may be employed.

**Figure 4:**
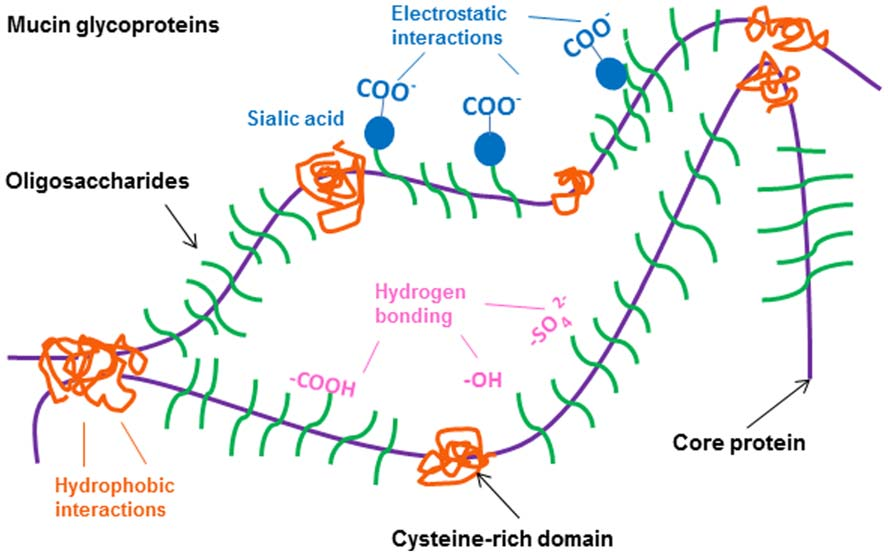
Schematic structure of mucin glycoproteins and their potentially mucoadhesive elements.

In the second part, we analyzed the mobility of negative, positive and PEGylated nanoparticles in porcine tracheal respiratory mucus and compared this to the motion characteristics of PRV. Mucin content, governed by mucin secretion rates as well as the degree of mucus hydration, is a major determinant of mucus rheology. Small differences in the concentrations of mucins may be sufficient to cause significant changes in the mucus viscoelasticity [37], [38]. Therefore, to retain the rheological properties of mucus, a small volume of PRV or nanoparticle suspension was mixed with porcine tracheal respiratory mucus. At the time scale of 1 s, PRV (−31.8±1.5 mV, 245.7±11.5 nm) experienced a significant obstruction in porcine tracheal respiratory mucus, with approximately 96% of the population exhibiting subdiffusive transport. The slightly smaller PEGylated nanoparticles (9.6±0.8 mV, 232.6±2.4 nm) exhibited a 160-fold higher <*MSD*> than PRV at the time scale of 1 s (Figure 2A). Our results indicate that the immobility of PRV in porcine tracheal respiratory mucus cannot be explained by a size filtering mechanism. Instead, a complex interplay of mucoadhesive interactions is proposed to fully explain this issue. Therefore, the mobility of positively charged and negatively charged nanoparticles, nearly neutral PEGylated nanoparticles and PRV was measured. The strongly charged nanoparticles and PRV were significantly trapped in porcine tracheal respiratory mucus, whereas relatively neutral PEGylated nanoparticles were diffusive, implying the role of electrostatic forces in the interactions between mucus and particles. Similarly, Lieleg et al. [39] analyzed the diffusions of amine-, carboxyl-terminated and PEGylated particles (1 µm) in extracellular matrix (ECM) purified from the Engelbreth-Holm-Swarm sarcoma of mice, and found that the diffusion of charged particles was extremely suppressed compared to the neutral PEG modified particles, indicating particle mobility was related to the surface charge. Moreover, it is reported that the higher the surface potential of amine-terminated particles, the stronger the particle mobility was suppressed in porcine gastric mucin, and that a high salt concentration could increase the mobility of amine-terminated particles by shielding the surface of particles with counter ions [40]. Our findings suggest that charged particles interacted with the mucin polymers via electrostatic forces, and these interactions may reduce particle mobility. Furthermore, the non-glycosylated protein regions may provide a sufficiently hydrophobic region for adequate particle-mucin interactions (Figure 4). This is supported by an investigation of the translocation of modified polystyrene microspheres in gastrointestinal mucin, in which the least hydrophobic amidine-terminated microspheres were found to have the highest diffusion among the microspheres [41]. In addition, the envelope of PRV and hydrophobic domains on its glycoproteins may be subjected to the hydrophobic cysteine-rich domains along mucin fibers, forming multiple low-affinity bonds. Although their low-affinity bonds have short half-lives and are easily broken by thermal energy [42], a great number of such bonds can keep virus particles attached to mucin fibers and entrap them. Lastly, hydrogen bonding appears to play a crucial role in mucoadhesion and disruption of hydrogen bonds can substantially reduce the adhesive strength of a mucoadhesive system. Previous studies showed that the addition of hydrogen bond breaking agents, such as urea and potassium thiocyanate (KCNS), to porcine gastric homogenized mucus resulted in a reduction in the elastic (G’) and viscous (G”) values [43] and that amylose gel strength could be significantly reduced by the addition of urea [44]. Interestingly, mucins contain a high density of negatively charged glycoproteins that present both strong proton acceptor and donor functionalities which mediate hydrogen bonding [45]. We thus hypothesize that sialic acid and sulphate residues on the oligosaccharide chains of the mucin glycoproteins (Figure 4) are likely to form hydrogen bonds with the glycoproteins of entrapped PRV particles. Therefore, investigating the influence of sialic acid and sulphate residues of porcine respiratory mucins on the mobility of PRV could help to further unravel the interplay between PRV particles and the mucus barrier.

In the third part, we modified the surface of PRV particles by PEGylation and measured the mobility of the resulting virus particles. Lysine, one of the most prevalent amino acids in proteins, is known as an ideal target for PEGylation. The NHS esters of mPEG-NHS react with the primary amine groups of lysine to form stable amide bonds. Lysine mediated PEGylation of adenoviruses [46] and vesicular stomatitis virus G pseudotyped lentivirus vectors [47] have been described to sufficiently shield the viral surface and to protect the viruses from immune responses. In our study, attachment of mPEG-NHS to the PRV surface significantly increased the mobility of the PEGylated PRV. After PEGylation, the virus still possessed a net surface charge of −13.8±0.9 mV (Table 2), thus implying that besides highly reduced charge-charge interactions, other important interactions may be abolished. The coverage of the surface of virus/nanoparticles with uncharged, hydrophilic PEG did not only neutralize the charges which mediates electrostatic interactions, but also shielded the hydrophobicity and capability of hydrogen bonding. Hence, PEGylation could turn the “active” PRV into more “inert” particles.

Alternatively, PRV may bind directly to specific components of the respiratory mucus. It is known for PRV that heparan sulfate acts as a cellular receptor for viral attachment and subsequent entry [48], [49]. In addition to the cell surface heparan sulfate, secretory heparan sulfate may be present as one of the major proteoglycans in the airway secretions [50], [51]. These heparan sulfates in different forms might function as a “captor” for PRV. We thus conducted experiments using polyclonal antibodies (hyperimmune serum) to determine if the entrapment of PRV in the porcine tracheal respiratory mucus could be mediated by binding to heparan sulfate (data not shown). Although it is not known if the PRV glycoprotein C (gC) was fully blocked, we assume this glycoprotein, responsible for heparan sulfate binding, to be blocked by the polyclonal antibodies. However, the transport rate of the neutralized PRV was nearly the same as that of the natural virus, implying that other interactions rather than specific PRV-receptor binding may play a determinant role in such virus-mucus interactions. Furthermore, to determine if mucins may bind PRV, we performed immunofluorescent stainings using MUC5AC antibodies that recognize the protein backbone of the mucin glycoproteins and PRV gC monoclonal antibodies (data not shown). Attachment of PRV to the mucin network was observed, but no colocalization was seen, suggesting that the virus did not bind to the protein backbones of the mucins. Therefore, it would be interesting to investigate if the sugar side chains of mucins can be a target.

Our findings have clearly shown that porcine tracheal respiratory mucus is able to act as a barrier against PRV infection. This might explain in part the pathogenesis of PRV, as no signs of viral replication are found in the trachea *in vivo*. Next to that, our laboratory used *in vitro* models of respiratory mucosae of different parts of the respiratory tract to study the invasion of different viruses through mucosal surfaces. When applying PRV on porcine tracheal mucosae, we found that PRV was able to strongly replicate in the epithelium and even to breach the basement membrane to infiltrate the host (Steukers et al., unpublished data). As these tissues were washed several times before inoculation with a virus, little mucus remained as a coating on the surface. These latter findings put strength to our hypothesis that porcine tracheal respiratory mucus might be responsible for protection against PRV infection in the proximal trachea. It would therefore be highly interesting to analyze the barrier properties of porcine nasal respiratory mucus against PRV and compare this to the results presented in this study. A comparison can also be made with another alphaherpesvirus in bovine. Similar as for PRV, bovine herpesvirus 1 (BoHV-1) replicates in the upper respiratory tract. However, this is not limited to nasal mucosae as BoHV-1 strongly replicates in the proximal trachea as well. This is confirmed by a study performed in our lab by using bovine respiratory mucosal explant models which show that different upper respiratory tissues are susceptible to BoHV-1 infections [2]. Extrapolation of our hypothesis for PRV to BoHV-1, suggests that bovine tracheal respiratory mucus might not have similar protective characteristics as the porcine counterpart and that this might be a matter of virus-host co-evolution. Further investigation on this matter might provide us some crucial information on the invasion capacities of different alphaherpesviruses through the mucus barrier. Additionally, an important aspect of mucus is that the gel phase mucus is a discontinuous layer. This is due to the fact that the mucus does not flow evenly but preferentially concentrates along troughs or grooves [52]. The thickness of rat tracheal mucus observed with light- and transmission electron microscopy ranged between 0.1 and 50 µm [11], indicating there were thin or even “bare” regions that may be more susceptible to viral insult. This might also explain why certain regions are more susceptible to PRV infection in the explant model. To fully illustrate this issue, further investigation will be needed.

In summary, we applied single particle tracking to study particle diffusion in porcine tracheal respiratory mucus, and developed an *in vitro* model to analyze the mobility of PRV in the mucus. We found that PRV was significantly trapped in porcine tracheal respiratory mucus, due to complex mucoadhesive interactions. Thus, mucoadhesion may play a significant role in the host defense. This model may be a useful tool in revealing the invasion mechanisms of alphaherpesviruses and in providing some novel insights into the strategies of mucosal immunity.
